# *Bufonid herpesvirus* 1 (BfHV1) associated dermatitis and mortality in free ranging common toads (*Bufo bufo*) in Switzerland

**DOI:** 10.1038/s41598-018-32841-0

**Published:** 2018-10-03

**Authors:** Francesco C. Origgi, Benedikt R. Schmidt, Petra Lohmann, Patricia Otten, Roman K. Meier, Simone R. R. Pisano, Gaia Moore-Jones, Marco Tecilla, Ursula Sattler, Thomas Wahli, Veronique Gaschen, Michael H. Stoffel

**Affiliations:** 1 Centre for Fish and Wildlife Health (FIWI), Department of Infectious Diseases and Pathobiology, Vetsuisse Faculty, University of Bern, Länggassstrasse 122, 3001 Bern, Switzerland; 2 Info fauna Karch, UniMail, Batiment G, Bellevaux 51, 2000, Neuchâtel, Switzerland; 3 Department of Evolutionary Biology and Environmental Studies, University of Zurich, Winterthurerstrasse 190, 8057 Zurich, Switzerland; 4 Veterinarian, 8127 Forch, Switzerland; 5 Fasteris SA, Geneva, Switzerland; 6 University of Milan, Milan, Italy; 7 Division of Veterinary Anatomy, University of Bern, POB, Bern, Switzerland

## Abstract

Here we report the discovery and partial characterization of a novel herpesvirus tentatively named *Bufonid herpesvirus* 1 (BfHV1) from severe dermatitis in free ranging common toads (*Bufo bufo*) in Switzerland. The disease has been observed in toads every year since 2014, in spring, during the mating season, at different and distant locations. The virus is found in the skin and occasionally in the brain of infected toads. The genome of the virus is at least 158 Kb long and contains at least 152 open reading frames with a minimal length of 270 nt. The genome of BfHV1 contains all the signature genes that are present in alloherpesviruses. Phylogenetic analysis based on the amino acid sequence of the DNA polymerase and terminase proteins positions the novel virus among the members of the genus *Batrachovirus*, family *Alloherpesviridae*. This is the first herpesvirus ever characterized in common toads.

## Introduction

Amphibians are undergoing a major decline worldwide^1,2^. The reasons for this unprecedented loss in biodiversity are only partially understood^3^. Among potential contributors, infectious diseases have recently raised attention as important players^4,5^. In addition to well-characterized amphibian pathogens including chytrids^6–9^ and ranaviruses^5,10,11^, it is likely that other pathogens or putative pathogens, whose role in amphibian disease ecology is basically unknown, though not irrelevant, are still to be discovered and better investigated^12^. Herpesviruses for example, have been long known in frogs^13,14^; however, they still remain under-investigated. Accordingly, during the last 25 years, a herpesvirus was repeatedly observed in European frogs^12,15,16^. However, *Ranid herpesvirus* 3 (RHV3) was finally identified and characterized only recently^17^. This virus is associated with proliferative skin disease, whose impact on the affected frogs is unknown but given the severe and extensive skin lesions is considered clinically relevant.

A similar, but distinct proliferative skin disease has recently been observed in free-ranging common toads (*Bufo bufo*) in multiple regions of Switzerland including a location where a mass mortality event occurred. The disease had been observed by us since 2014; after volunteers patrolling amphibian migrations to the breeding sites between the late winter and early spring reported that about 10% of the observed toad populations were affected. Interestingly, the common toad populations both in Switzerland and in UK have been reported to undergo a negative trend and the reasons for this are currently unknown^18^.

Here we report the results of a study aiming to partially characterize this emerging disease in free-ranging toads and its associated viral agent, tentatively named *Bufonid herpesvirus* 1 (BfHV1). This is another disease that, similar to RHV3-associated skin disease, may have been overlooked or underestimated in previous years, but that is likely to have been present in the free-ranging European common toad population and might have been associated with mortality.

## Material and Methods

### Animals

From 2014 to 2017, a total of 48 common toads (n = 1 in 2014, n = 3 in 2015, n = 1 in 2016 and n = 43 in 2017), collected in different locations (cantons of Basel-Landschaft, Zurich and Neuchatel) in Switzerland, were examined at the Centre for Fish and Wildlife Health (FIWI) of the University of Bern (Table 1). The first individuals with skin lesions were received in 2014 (n = 1) and in 2015 (n = 3). They were all found alive and sampled in the region of Rothenfluh, canton of Basel-Landschaft. In 2016, another toad was seen alive, but lethargic and with obvious skin lesions in a pond in Eichbuehl (Eichbuehl, E-pond-ZH) in the canton of Zurich and was collected. In 2017 a total of 43 toads were collected. Forty one of them were collected in a pond in canton of Zurich (Forch = F-pond-ZH); one in another pond in the same canton (Affoltern = A-pond-ZH) and another one in a pond in canton of Neuchatel (Combacervey). Of the 41 toads collected from the F-pond-ZH, 33 were found dead, whereas the remaining 8 were alive but displayed poor body condition. The toad sampled at the A-pond-ZH, was also found alive but in poor body condition. A die-off of common frogs (*Rana temporaria*) was also occurring in this pond at the time of sampling and five frog carcasses were also collected for examination. Finally, the last toad received in 2017 was collected alive in a pond in the canton of Neuchatel, where several animals showed skin lesions but no dead animals were seen. All the toads were collected each year between the end of March and beginning of April, during the mating season. Collection of toads was authorized by the local nature conservation authorities for diagnostic purposes and no additional permits were required.

**Table 1:** Toad sampling summary.

Canton	Location	Status	2014	2015	2016	2017
BL*	Rothenfluh	Alive	1 (1;1)^a^	3 (3;3)^a^		
ZH*	Pond E**	Alive, lethargic			1 (1;1)^a^	
	Pond F**	Alive, poor condition				8 (0;5)^a^
		Dead				33 (8^b^;7^c^)^a^
	Pond A**	Alive, poor condition				1 (0;0)^a^
NE*	Combacervey	Alive				1 (1;1)^a^
						43 (14;18)^a^

In the table below are shown the total number of toads collected during the study with their origin and time of sampling along with the gross skin lesions and viral DNA detection results.

*BL: canton of Basel-Landschaft; ZH: canton of Zurich; NE: canton of Neuchatel.

**E: Eichbuehl, canton of Zurich; F: Forch, canton of Zurich; S: Affoltern, canton of Zurich.

^a^The superscript letter refers to the number of toads showing gross skin lesions (X) and positive by PCR (Y) for BfHV1 genomic DNA arranged in the parenthesis above as follows: (X, Y).

^b^Of these eight individuals, all showing gross skin lesions, seven ^c^were positive by PCR for the presence of BfHV1 genomic DNA, whereas one was negative.

### Pathology

All toads submitted alive were humanely euthanized with an overdose MS222 (Sigma; Buchs, CH) according to guidelines for use of live amphibians and reptiles in field and laboratory research (https://www.aaalac.org/accreditation/RefResources/SS_Amphib.pdf) and fully necropsied. No institutional and/or licensing committee approval was necessary because the euthanasia was carried out for diagnostic and not for experimental purposes. Tissue samples were collected, formalin fixed (10%), processed, embedded in paraffin, sectioned at 5 μm and stained with hematoxylin and eosin according to routine protocols. Additional samples of skin, kidney and liver were both fixed in 2.5% glutaraldehyde and embedded for transmission electron microscopy (TEM) or frozen at −80 °C for further analysis.

### Electron Microscopy

The skin samples selected for Electron Microscopy were those showing obvious gross and histological lesions. Skin samples were processed as reported previously^17^ and examined with a Philips CM12 transmission electron microscope (FEI, Eindhoven, Netherlands).

### Virus Isolation

Virus isolation was carried out according to an established protocol^17^. Skin tissue homogenates were used to obtain the inoculum. The inoculum was seeded onto TH-1 cells (Subline B1; -ATCC^®^CCL50™), ICR-2A (Istituto Zooprofilattico Sperimentale della Lombardia e dell’Emilia, cell line repository), and on primary common toad kidney cells which were then incubated at 17 °C (ICR-2A) and/or 25 °C (ICR-2A, TH-1, primary toad kidney cells), and monitored daily for cytopathic effects (CPE) for 4 weeks. Presence of virus was also tested by PCR (see detailed protocol below) in case the virus would have not been cytolytic and no CPE would have formed.

### Next generation sequencing

Three micrograms of total DNA pooled from that extracted from the skin sections, associated with obvious lesions, of the three toads collected in 2015 were delivered to a biotechnology company (Fasteris SA, Switzerland) for next generation sequencing (NGS). The DNA template library was sequenced with the Illumina HiSeq2500 to obtain 125 base long paired-end reads. *De novo* genome assembly was performed using the VELVET software (Version 1.3; http://www.ebi.ac.uk/zerbino/velvet/) as reported previously^17,19^. The paired Illumina reads were mapped to the obtained assembly using BWA^20^; the mapping was visualized with IGV (Integrative Genomic Viewer 2.3.34)^21^. A final consensus sequence was obtained by mapping the reads on the assembled sequence using the software Geneious (V.91.5; http://www.geneious.com)^22^ with standard setting and 25 iterations. Questionable assembly regions were further assessed by conventional PCR and Sanger sequencing. For open reading frame (ORF) identification, the software “ORF-finder” (https://www.ncbi.nlm.nih.gov/orffinder/) was used and an arbitrary cutoff of at least 90 codons was elected. Each of the amino acid (aa) sequences encoded by the predicted ORF was “blasted” with the program “BLASTp“ (http://blast.ncbi.nlm.nih.gov/Blast.cgi). All the predicted aa sequences that yielded no results with BLAST were examined with the software “Phyre2” (http://www.sbg.bio.ic.ac.uk/~phyre2/html/page.cgi?id=index) for specific domain recognition and structural modeling.

### Molecular diagnostics

A PCR protocol was developed based on the nucleotide sequence of the DNA polymerase gene obtained by NGS (see above). More specifically, 40 pMol each of a forward (5′-CAAACTGAAGATACAGAAGGTTGGGG-3′) and a reverse (5′-GCGCGAGTGCTTTGTACGCAACC-3′) primer were added to a mix composed of 3 μl of 10X reaction buffer, 0.4 μl of a 10 mM dNTPs mix, 5 units of Taq DNA polymerase (Qiagen, Hombrechtikon, CH) and deionized-distilled water to a final volume of 30 μl. The cycling reaction was carried out on a thermocycler 9600 Fast (Applied Biosystem, Foster City, CA) and consisted of an initial denaturation step at 95 °C for 3 minutes followed by 35 cycles comprising a 30 seconds denaturation step at 95 °C, 30 seconds annealing at 55 °C and an elongation step at 72 °C for 30 seconds. A final elongation at 72 °C for 10 minutes was carried out to exhaust the polymerase. The obtained amplicons were resolved in a 2% agarose gel and examined under UV light.

Established protocols for the detection of ranavirus^23^, Batrachochytrium dendrobatidis (Bd)^24^ and RHV3^17^ were also carried out on all the toads collected in 2017.

### Sanger Sequencing

The PCR amplicons were sequenced using the same primers as for their amplification with an automated sequencer (ABI Prism 3100 genetic analyzer; Applied Biosystems, Foster City, CA) using the BigDye Terminator cycle sequencing kit (Applied Biosystems, Foster City, CA) according to the manufacturer’s instruction.

### Phylogenetic analysis

The phylogenetic analysis was carried out by comparing the aa sequence of the DNA polymerase and of the terminase proteins of the novel herpesvirus with the homologous sequences from other known alloherpesviruses similarly to how described previously^17^. In particular the aa sequences of the DNA polymerase protein encoded by 10 *Alloherpesviridae* were compared to the homologous protein encoded by the BfHV1 genome (*Bufonid HV1*- MF143550; *Ictalurid HV1*-NP_041148.2; *Ictalurid HV2*-ACZ55873.1; *Acipenserid HV2*-ACZ55868.2; *Ranid HV2*-ABG25576.1; *Ranid HV1*-YP_656727.1; *Ranid HV*3-KX832224; *Anguillid HV1*-ADQ54121.1; *Cyprinid HV1*-AAX53084.1; *Cyprinid HV2*-YP_007003898.1; *Cyprinid HV3*-AAX53082.1). Furthermore, the aa sequence of the terminase protein encoded by eight alloherpesviruses was compared to the homologous sequence encoded by BfHV1 (*Bufonid HV1*- MF143550; *Ictalurid HV1*-NP_041153.2; *Ranid HV1*-YP_656697.1; *Ranid HV2*-YP_656576.1; *Ranid herpesvirus* 3-KX832224; *Anguillid HV1*-YP_003358149.1; *Cyprinid HV1*-YP_007003702.1; *Cyprinid HV2*-YP_007003857; *Cyprinid HV3*-YP_001096069.2). The aa sequence of the DNA polymerase and the concatenated aa sequences of the two putative exons of the terminase protein of *Testudinid herpesvirus 3* (TeHV3), an alphaherpesvirus, were selected as outgroup for the DNA polymerase- and terminase-based analysis, respectively. The aa sequences of the terminase protein of RHV3 and BfHV1 were also obtained by concatenating the putative coding exons. The aa sequences were aligned with ClustalW (SDS-Biological workbench; http://workbench.sdsc.edu) and a consensus maximum likelihood phylogenetic tree was constructed using the software Mega 6.0.6^25^ with the standard settings and 500 bootstrap replications, Jones Taylor Thorton (JTT) replication method, uniform rates among sites and very strong branch swap filter.

## Results

### Animals and pathology

#### Toad with gross skin lesions

**Figure 1:**
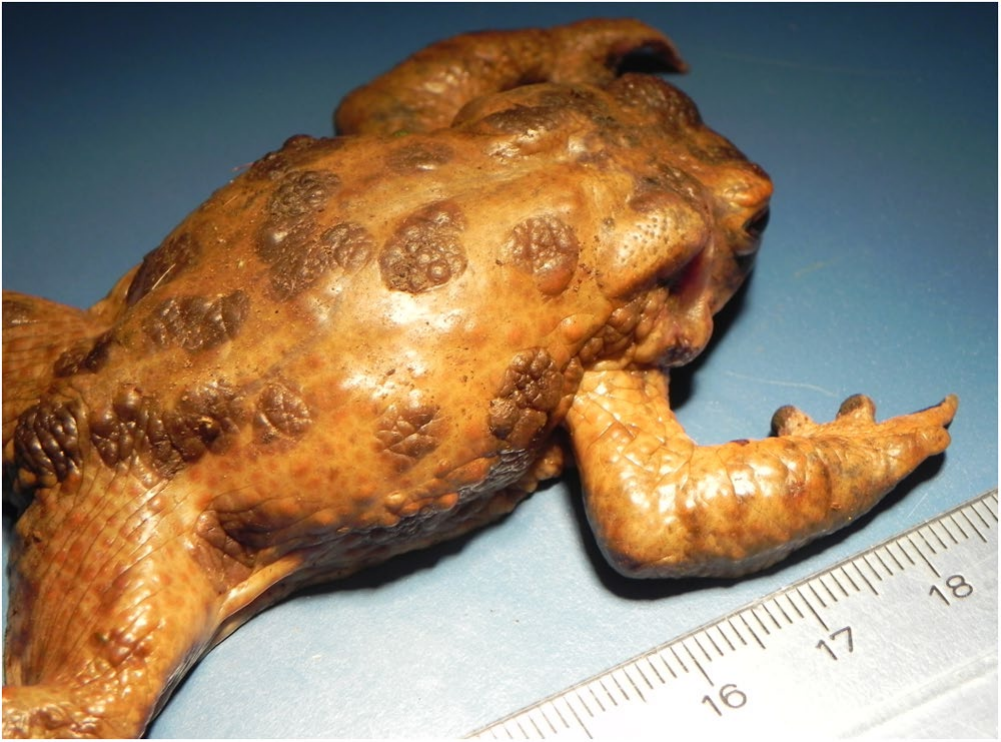
*Bufonid herpesvirus* 1 infected common toad (*Bufo bufo*) associated gross skin lesions. Multifocal raised, dark brown, patchy areas of skin are scattered over the dorsum of the toad.

**Figure 2:**
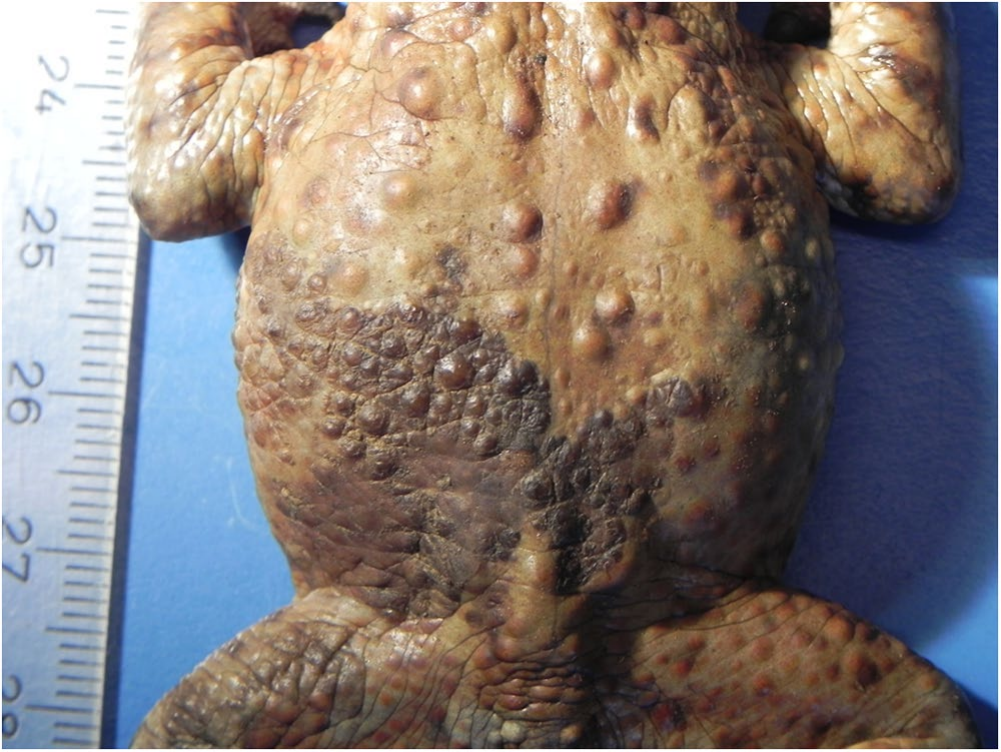
*Bufonid herpesvirus* 1 infected common toad (*Bufo bufo*) associated gross skin lesions. Occasionally, coalescing skin proliferations form extensive lesions.

**Figure 3:**
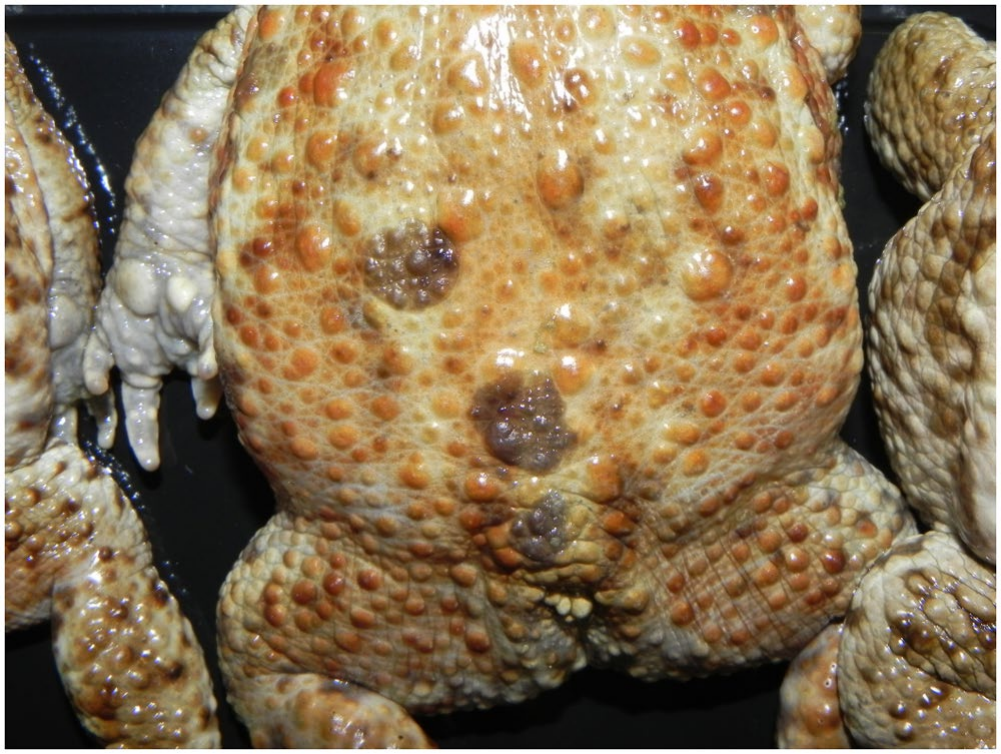
*Bufonid herpesvirus* 1 infected common toad (*Bufo bufo*) associated gross skin lesions. Round, moderately raised and isolated skin proliferations are observed in this toad.

**Figure 4:**
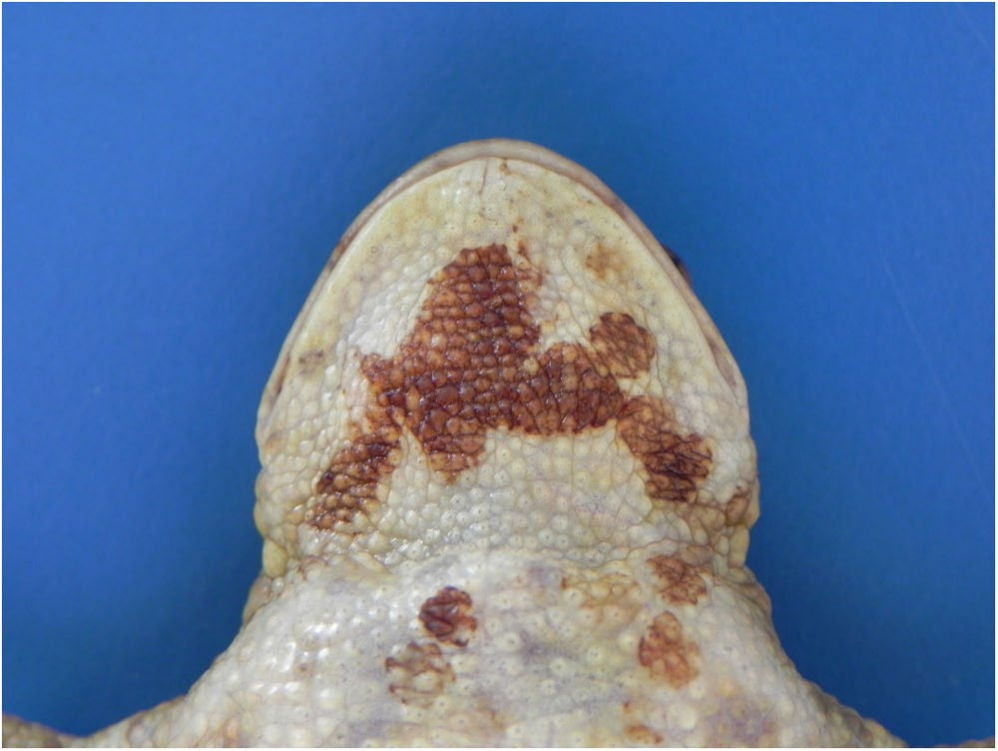
*Bufonid herpesvirus* 1 infected common toad (*Bufo bufo*) associated gross skin lesions. Abnormal skin shedding and retained skin is observed over the gular region of this toad.

Fourteen out of 48 toads examined during this investigation showed consistent skin lesions. These included all the five toads collected in 2014, 2015, 2016 and nine out of the 43 toads collected in 2017 (Table 1). All the toads with lesions collected in 2014, 2015, 2016 and one of those collected in 2017 (canton of Neuchatel) were alive. The other eight toads with skin lesions from 2017 (F-pond-ZH) were found dead. Grossly, the skin was characterized by prominent, raised, round, brown multifocal to confluent patches (Figs 1–4). Abnormal skin shedding and retained skin was commonly observed in the affected skin areas (Fig. 4). Presence of lung nematodes (*Rhabdias* sp) was common. No additional gross changes were observed.

**Figure 5:**
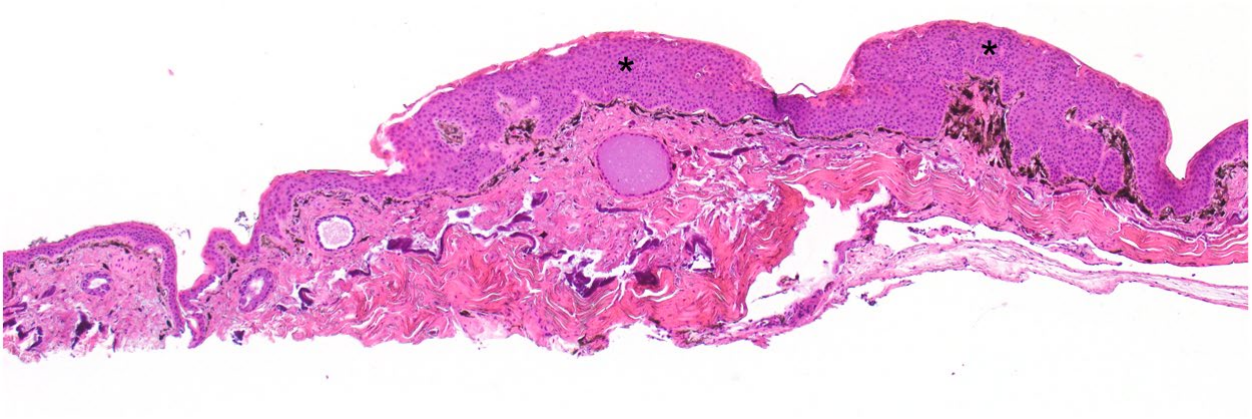
*Bufonid herpesvirus* 1 infected common toad (*Bufo bufo*) associated microscopic skin lesions. The raised skin lesions correspond to areas of epidermal hyperplasia (asterisks).

**Figure 6:**
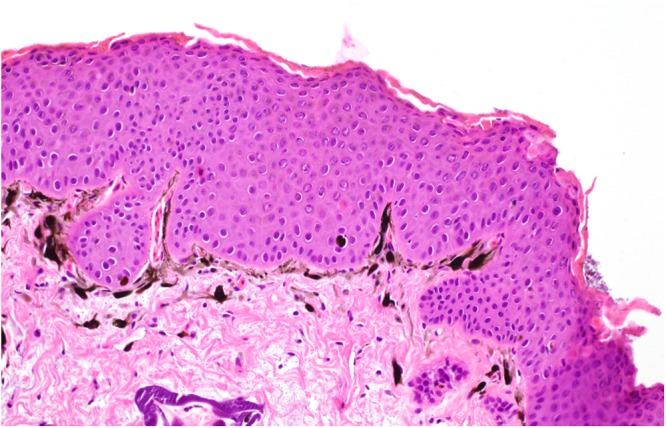
*Bufonid herpesvirus* 1 infected common toad (*Bufo bufo*) associated microscopic skin lesions. The affected epidermal layer is thickened up to five times normal.

**Figure 7:**
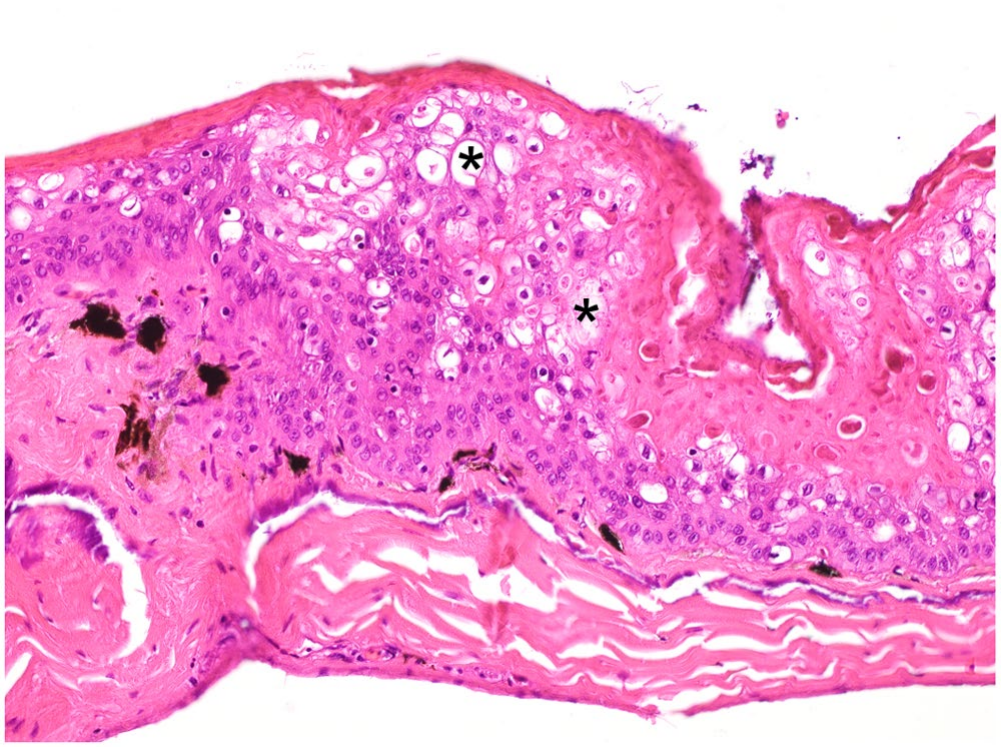
*Bufonid herpesvirus* 1 infected common toad (*Bufo bufo*) associated microscopic skin lesions. Variable cytoplasmic vacuolization (asterisk) is occasionally observed in the affected epidermis.

**Figure 8:**
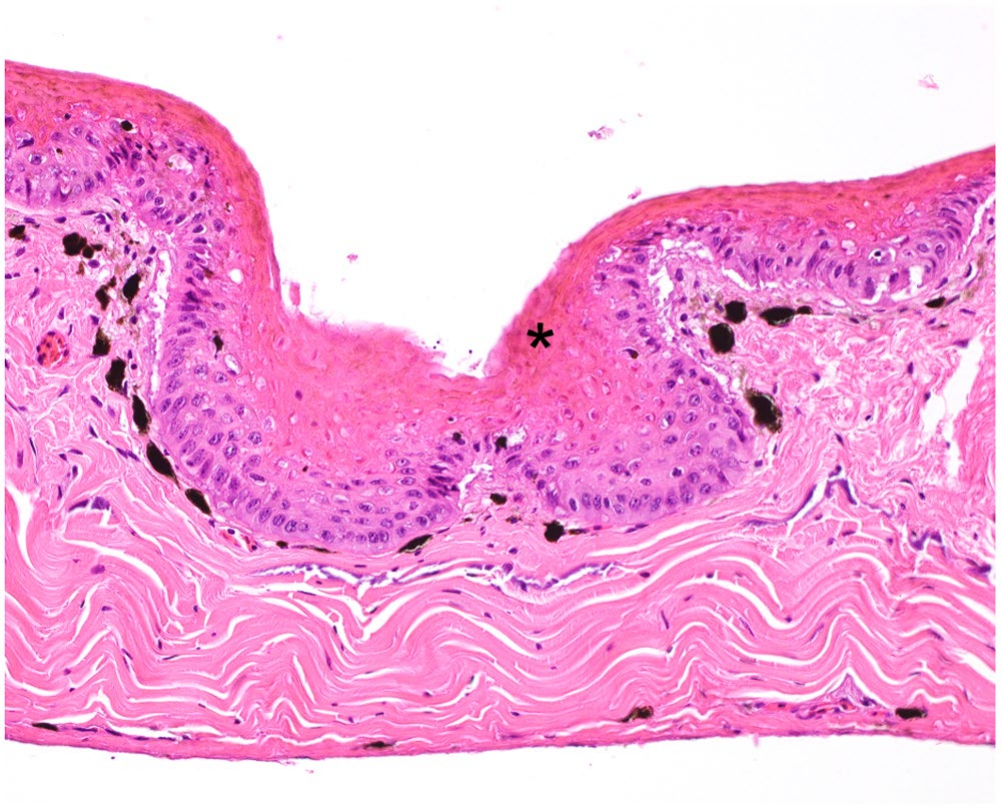
*Bufonid herpesvirus* 1 infected common toad (*Bufo bufo*) associated microscopic skin lesions. Affected areas of the epidermis show prominent and exuberant keratinization (asterisk).

**Figure 9:**
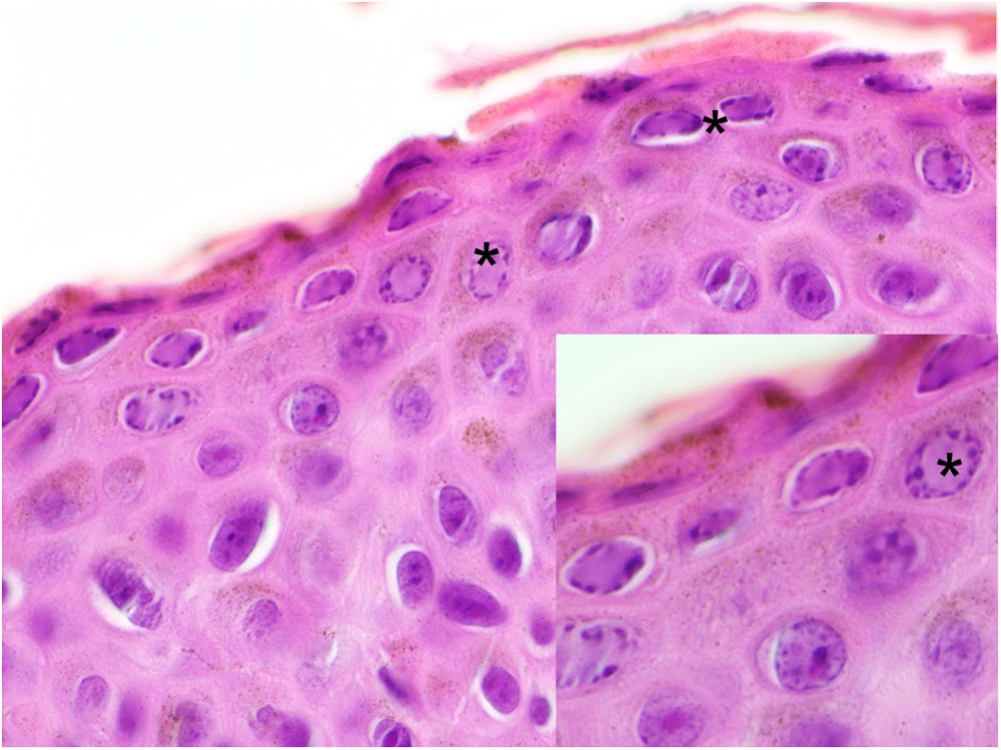
*Bufonid herpesvirus* 1 infected common toad (*Bufo bufo*) associated microscopic skin lesions. In the hyperplastic epidermis, moderate numbers of intranuclear eosinophilic to amphophilic inclusions (asterisk) are found (main figure and inset).

Histologically, the brown skin patches corresponded to areas of moderate to severe nodular to plaque-like epidermal hyperplasia up to five times normal (Figs 5–9). The epidermal lesions were predominantly affecting the *stratum spinosum* and only occasionally the *stratum basale*. The affected epidermal areas showed exuberant epithelial proliferation, intercellular edema (spongiosis), intracytoplasmic and intranuclear vacuolization (Fig. 7) along with prominent parakeratosis and dyskeratosis (Figs 7 and 8), though not consistently. Few to large numbers of intranuclear eosinophilic to amphophilic inclusions were frequently observed in the keratinocytes of the affected skin (Fig. 9 and inset). Some individuals showed intracytoplasmic eosinophilic to amphophilic droplets as well. Intralesional inflammatory infiltrates were rarely observed at the interface between the *stratum basale* of the epidermis and the underlying dermis. No other histological changes were observed in the examined tissues.

#### Toad without gross skin lesions

Skin lesions similar to those described above were not observed in the remaining 34 toads, which were all collected in canton of Zurich in 2017. These included 25 of the 33 dead individuals and the 8 toads collected alive, all from the F-pond-ZH and the single toad collected alive in the A-pond-ZH. The toads did not show significant gross changes except for the presence of *Rhabdias* sp in their lungs. Dead common frogs collected in the A-pond-ZH showed advanced autolytic changes in absence of detectable gross lesions.

Histologically, minimal to mild epidermal hyperplasia was rarely observed. Mild to moderate vacuolization of the epidermis was observed in two of the batch of 25 toads from the F-pond-ZH. However, the severity of the histological changes was inconspicuous when compared to those observed in the toads with severe macroscopic skin lesions. A mild encephalitis was observed in one of the 8 toads collected alive in the F-pond-ZH. No additional changes were observed.

The frogs collected in the A-pond-ZH showed severe and extensive autolysis. The epidermal layer of the skin was showing end-stage autolytic changes in the examined sections.

### Electron Microscopy

**Figure 10:**
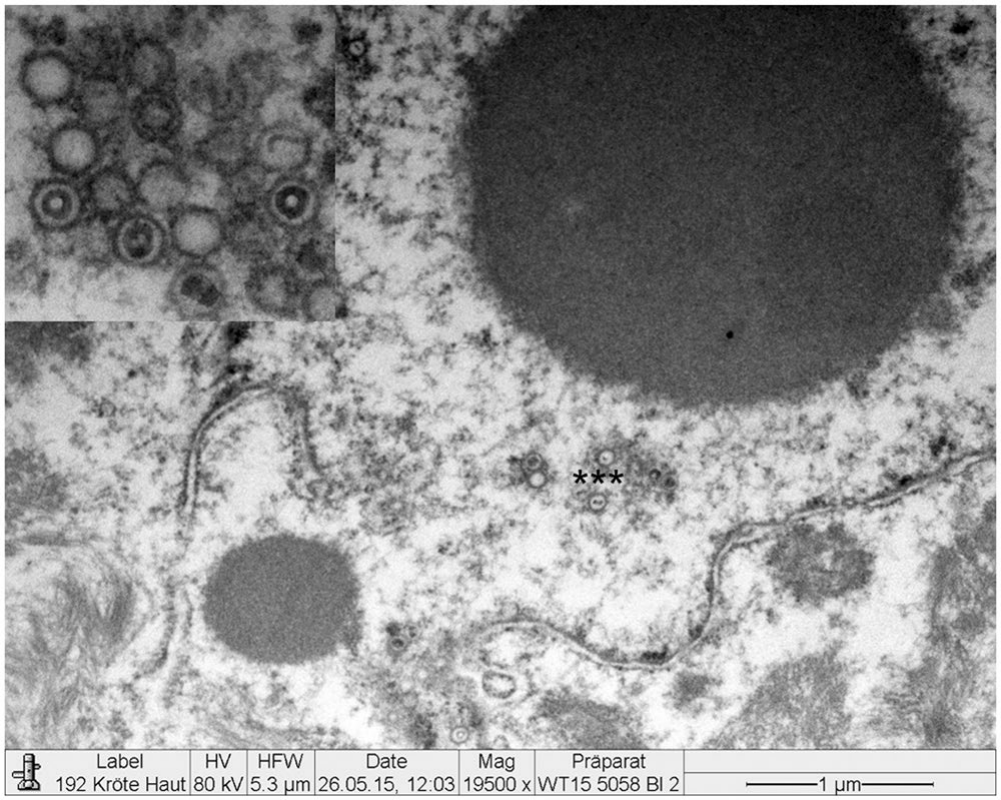
*Bufonid herpesvirus* 1 infected common toad (*Bufo bufo*) associated ultrastructural skin lesions (Transmission Electron Microscopy-TEM). A cluster of naked viral particles with icosahedral symmetry (asterisk) are shown in the nucleus of one of the affected keratinocytes. The inset shows a higher magnification of the virions.

**Figure 11:**
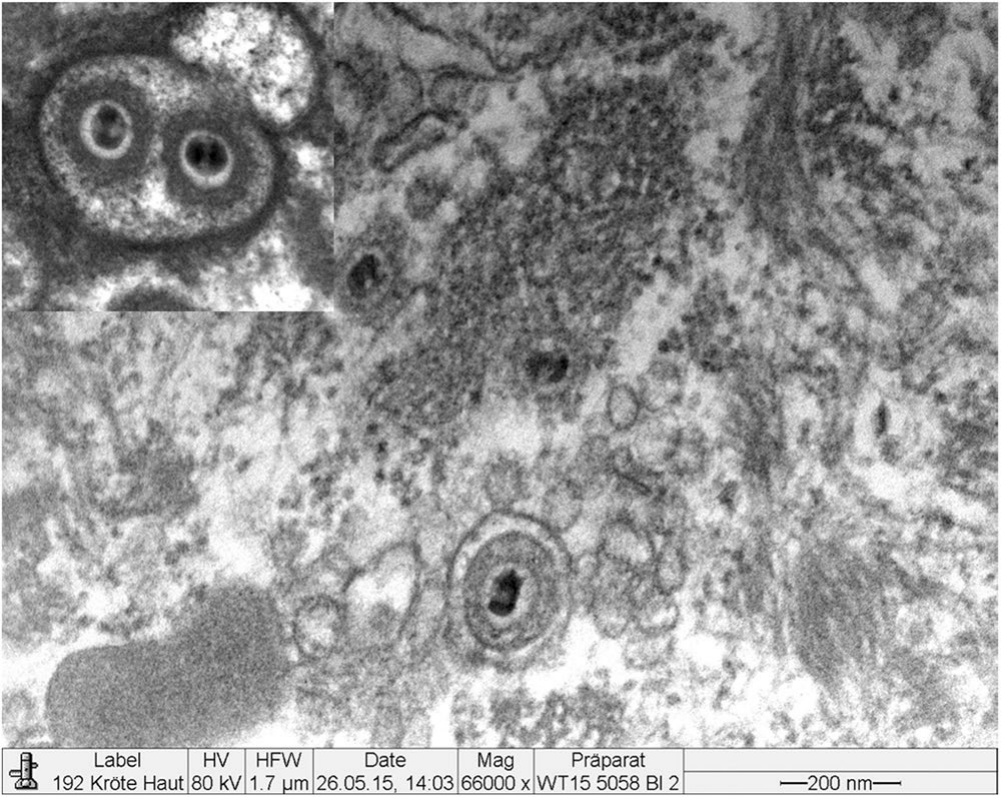
*Bufonid herpesvirus* 1 infected common toad (*Bufo bufo*) associated ultrastructural skin lesions (Transmission Electron Microscopy-TEM). Viral particles are surrounded by additional layers consistent with an integument and an envelope. Two particles are shown within the same envelope (inset).

**Figure 12:**
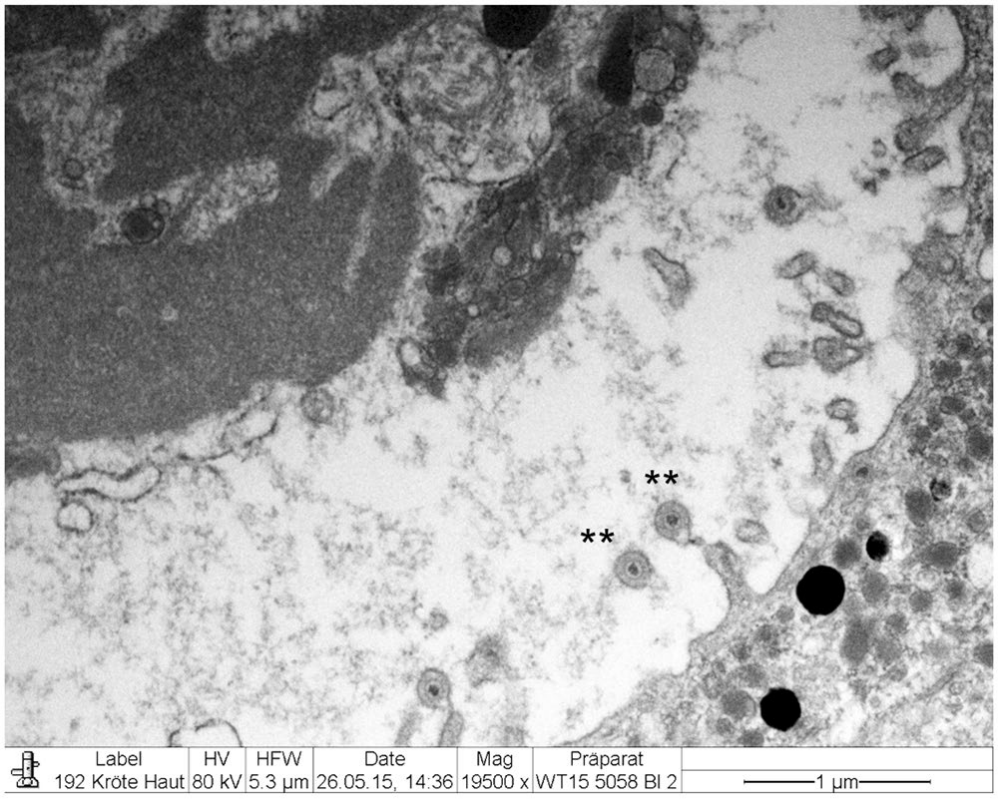
*Bufonid herpesvirus* 1 infected common toad (*Bufo bufo*) associated ultrastructural skin lesions (Transmission Electron Microscopy-TEM). Budding viral particles are observed in the image (asterisks).

**Figure 13:**
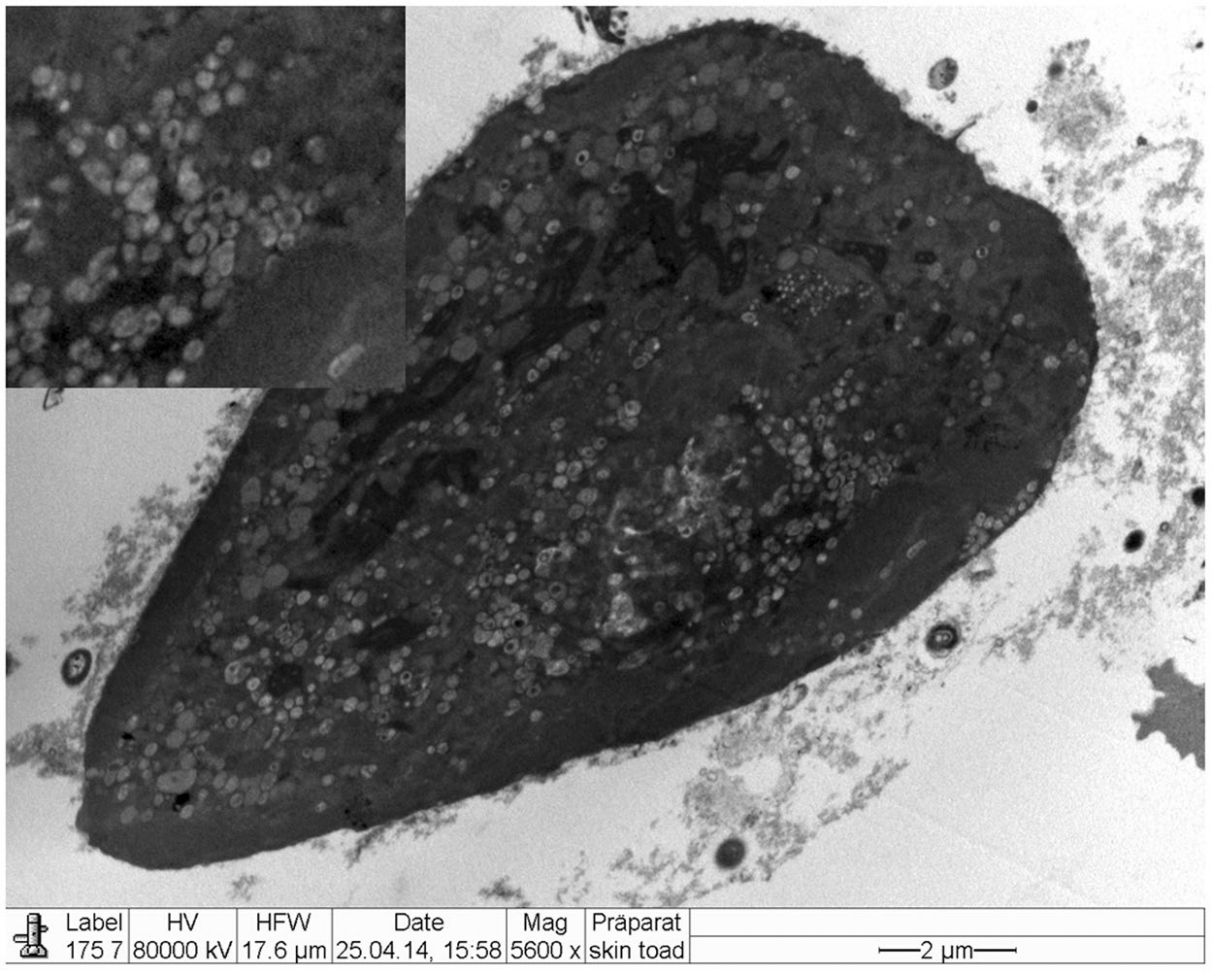
*Bufonid herpesvirus* 1 infected common toad (*Bufo bufo*) associated ultrastructural skin lesions (Transmission Electron Microscopy-TEM). A necrotic cell that sloughed from the affected skin contains large number of viral particles (inset).

Electron microscopy revealed the presence of intranuclear, electron dense particles with icosahedral symmetry measuring approximately 80 nm in diameter (Fig. 10). Outside the nucleus, similar particles were present but here they were surrounded by two concentric electrondense layers consistent with a herpesviral-like integument and an envelope that increased the particle size up to 120–150 nm (Fig. 11). Several viral particles budding from the membranes of the infected keratinocytes were observed (Fig. 12). Large numbers of viral particles were observed in the sloughing necrotic epithelial cells (Fig. 13).

### Virus Isolation

No virus could be isolated despite several attempts and multiple blind passages. None of the two cells lines and the primary toad kidney cells supported the growth of BfHV1 at the conditions selected for the virus isolation attempt. Furthermore, PCR reactions carried out on DNA extracted from the cells harvested during each of the blind passages failed to reveal the presence of BfHV1 genomic DNA.

### Molecular diagnostics

The specific BfHV1 PCR protocol developed for this investigation yielded an amplicon 255nt long correspondent to the partial sequence of the DNA polymerase gene. The PCR reaction using skin DNA as a template was positive in all five individuals showing proliferative dermatitis collected in 2014, 2015 and 2016. Of those collected in 2017, all but one of those showing gross skin lesions (n = 8) were also positive by PCR along with five of the 9 individuals not showing gross skin changes that were collected alive. Additionally, one of the toads with PCR positive skin showed a PCR signal from the brain. Positive PCR reactions were also obtained from the pooled DNA extracted from paraffin blocks where multiple organs (that did not include skin and brain) were embedded. Sanger sequencing carried out on selected samples confirmed the nature of the amplicons as part of the BfHV1 DNA polymerase gene. Ranavirus, RHV3 and Bd could not be detected by PCR in all the amphibians sampled in 2017, including the dead frogs collected in the A-pond-ZH.

### Next generation sequencing

Next generation sequencing yielded a total of 12′871 Mbases obtained with 51.4M passing filter clusters. The *de novo* assembly yielded a total of 257 scaffolds spanning from 241 to 110,936 nt for a total of 325′484 bases and a N50 of 4′690. Two scaffolds of 110 and 25 KB, respectively that encoded for putative proteins sharing similarities with those of ranid herpesviruses were selected to carry out the BfHV1 genome assembly. Following a 25-time iteration (Geneious (V91.5; http://www.geneious.com)^22^, overlapping sequences between the two scaffolds were identified and a final unique consensus circular sequence 158,250 bases long was obtained. The circular nature of the BfHV1 genome was confirmed by Sanger sequencing similarly to what described for RHV3^17^. This could represent a concatemer (as formed during replication) of the most likely linear form of the genome as known for all the other alloherpesviruses. Additional investigations are warranted to better define these features of the BfHV1 genome along with the conclusive identification of the putative genome ends. The average coverage per base was 2,004. The GC content is 40.59%.

### *Bufonid herpesvirus* 1 genome features

The BfHV1 genome was determined to be at least 158,250 nt long (Genbank accession number MF143550). It contains a total of at least 152 predicted ORFs encoding for at least 90 codons. The predicted size of the putative ORFs ranges from 270 nt (ORF53a) to 6,336 nucleotides (ORF65) (Table S1). Fifty-one of the predicted ORFs encode for hypothetical proteins sharing variable similarity with the homologous proteins of either *Ranid herpesvirus* 1 (RHV1) or - 2 (RHV2). ORF39 encoded for the putative ATPase subunit of the terminase, which showed the highest similarity (69%, Table S1). All the 12 genes conserved across all the alloherpesviruses have been identified in the genome of BfHV1 (along with the 13^th^ conserved gene common to all known alloherpesviruses except *Anguillid herpesvirus* 1) (Table S1). Of interest is the presence of a predicted ORF (ORF13) encoding putative proteins showing remarkable similarity with Epstein-Barr virus (EBV) gp42 (99% confidence on a 56% length coverage). Few C-type lectin encoding genes (including ORFs 4, 18, 19 and 21) have been identified along with ORF97 encoding for a putative member of the tumor necrosis factor (TNF) receptor superfamily (Table S1).

### Questionable genome regions

Multiple regions containing repeat regions were observed in the genome of *Bufonid herpesvirus* 1. Mapping of the reads against these regions showed abrupt coverage variations possibly suggesting assembly errors. A total of 13 regions with these features were identified and primers amplifying sequences spanning across each of these regions were designed and PCRs were carried out. All the PCRs yielded detectable bands consistent with a correct genome assembly. Six out of the 13 PCRs yielded an amplicon consistent with the expected size whereas the remaining seven PCRs showed differences in comparison with the expected amplicon length ranging from 50 to 500 bp. One of the PCR products was approximately 280 bp long and it was amplified from a region that was predicted to contain multiple repeats with an expected size ranging from 278 to 298 bp. The discrepancies between the obtained and expected amplicon sizes were interpreted as most likely secondary to variations in the number of repeat motifs of each specific region and not investigated further.

### Genome comparison

**Figure 14:**
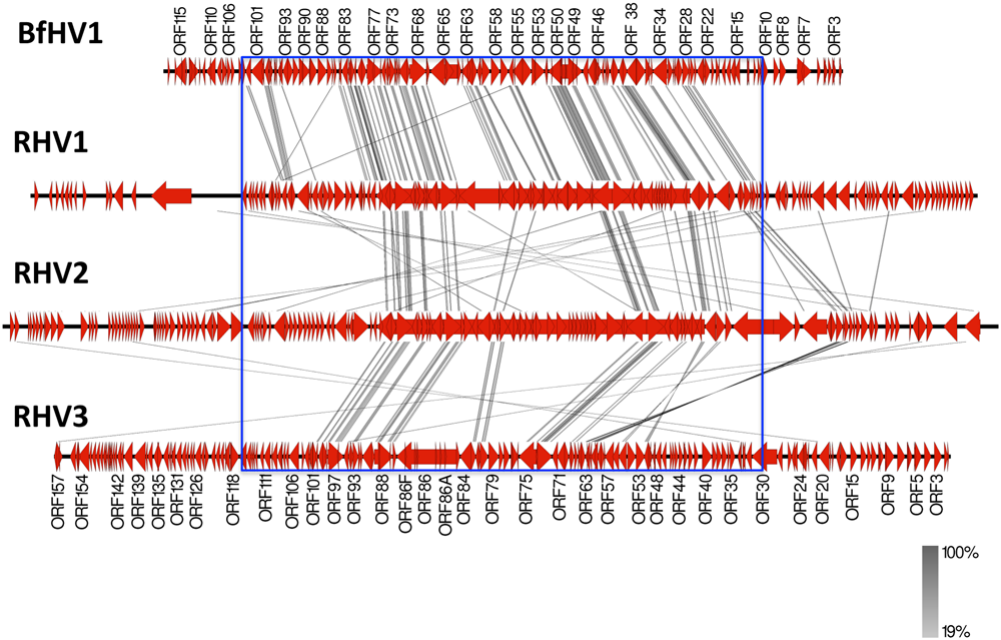
Genome Comparison (encoded proteins). The genome outlines of all the known RHVs along with that of the newly described BfHV1 are compared in this image. Connector lines link together conserved homologous encoded  proteins from the different genomes. The most conserved  proteins are encoded within the central portions of the genomes (blue rectangle). The intensity of the color of the connector lines (gray) is directly proportional to the degree of the conservation of the homologous encoded protein (the figure was obtained with the software Easyfig. 2.1; http://mjsull.github.io/Easyfig/ -See also ref.^26^).

Comparison of the BfHV1 genome (encoded proteins) with those of RHV1, -2 and -3 was carried out with Easyfig^26^. The connector lines in the diagram shown in Fig. 14 reveal that most of the conserved proteins are encoded within the central portion of the genomes as commonly observed in herpesviruses thus indirectly supporting the validity of the proposed BfHV1 genome assembly (Fig. 14).

### Phylogenetic analysis

**Figure 15:**
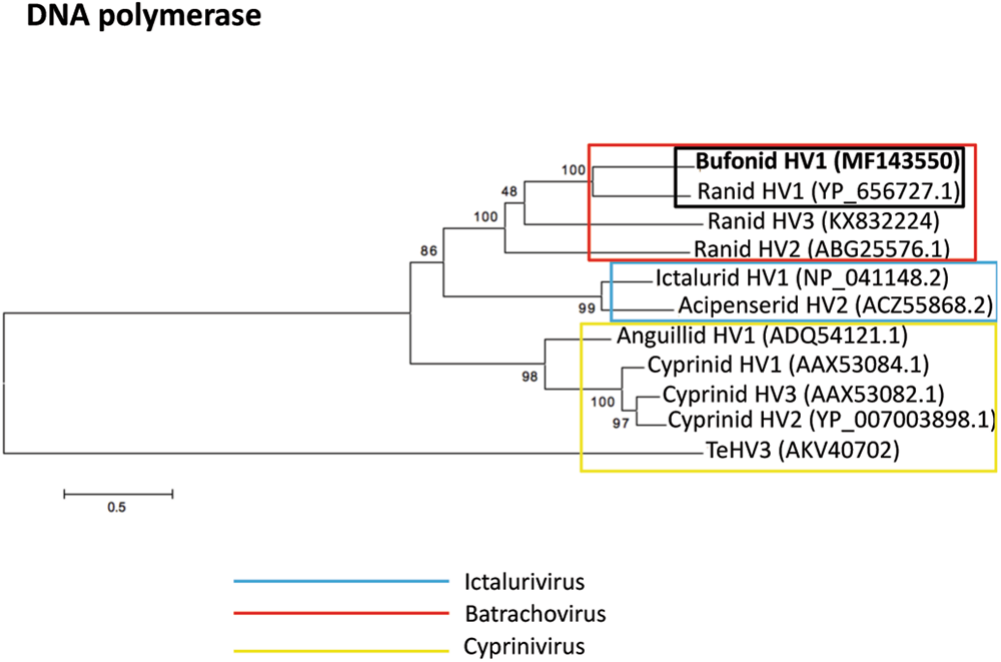
Phylogenetic analysis. The maximum likelihood tree based on the DNA polymerase amino acid sequence shows a clear clustering of BfHV1 among the members of the genus *Batrachovirus*. BfHV1 appears to be phylogenetically closer to RHV1 than to any other RHVs (the amino acid sequence of the TeHV3 DNA polymerase has been used as outgroup for the analysis); the Genbank accession numbers of each sequence used for the analysis are indicated in parentheses.

**Figure 16:**
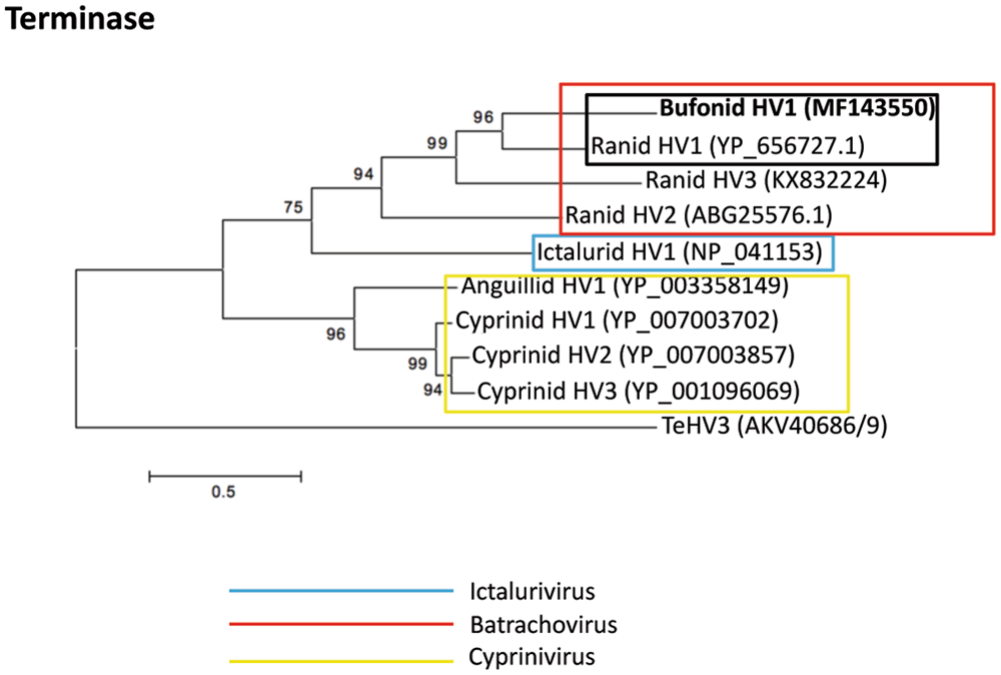
Phylogenetic analysis. The maximum likelihood tree based on the terminase amino acid sequence is similar to that shown in Fig. 15, and is supported by stronger bootstrap values (the amino acid sequences of the TeHV3 terminase has been used as outgroup for the analysis); the Genbank accession numbers of each sequence used for the analysis are indicated in parentheses.

The phylogenetic analysis showed unambiguous clustering of BfHV1 together with the members of the genus *Batrachovirus* (Figs 15 and 16). Convincing bootstrap values support the closer relatedness of BfHV1 with RHV1, which appear to separate also from RHV2 and RHV3, possibly suggesting the existence of distinct genogroups within the genus *Batrachovirus* (Figs 15 and 16).

## Discussion

Starting in 2014, free ranging common toads with a proliferative dermatitis were observed every year, with a peak in 2017, in Switzerland. The toads originated from different regions of the country and were initially found alive and clinically healthy. In 2017, however, affected toads were also found dead. Interestingly, dead toads were reported in the same pond also the year prior but they were not available for investigation. It is not clear if, and to what extent the skin disease contributed to the death of the toads but the observed lesions showed a broad range in severity, and given the decisive physiological role of skin in amphibians, a clinical relevance of the skin disease cannot be excluded. Common toads are one of the most widespread and abundant amphibian species in Switzerland and in Europe and they are currently not listed as a species of concern. However, a recent investigation revealed that common toads are undergoing a steady decline both in Switzerland and in the UK since the 1970s, calling attention to this species and its conservation status^18^. Independently of the number of toads with obvious skin lesions that we have observed and investigated during these years, is likely that these numbers are only a small fraction of the actual affected toad population. Accordingly, a toad collected in 2017 in a pond in canton Neuchatel was one of many showing the skin lesions consistent with the disease. A thorough epidemiological investigation is needed to better assess the current magnitude of the problem and the meaning of this proliferative skin disease of common toads in the greater context of amphibian disease ecology. The disease has invariably been associated with a novel herpesvirus that we tentatively named *Bufonid herpesvirus* 1 in absence of any other detectable agent or known pathogen. However no causative association between BfHV1 and the observed toad proliferative dermatitis has been proven at the moment. Fulfillment of Koch’s postulates will be necessary to prove any causality between the intralesional presence of BfHV1 and the observed proliferative skin disease in common toads.

*Bufonid herpesvirus* 1 has been detected by histology, electron microscopy and PCR. The virus is characterized by a genome containing several genes showing variable similarity with those of ranid herpesviruses. The molecular features of the virus are consistent with BfHV1 being a novel member of the genus *Batrachovirus* in the family *Alloherpesviridae*. Interestingly, the phylogenetic analysis clusters BfHV1 together with RHV1 as a monophyletic group, whereas RHV2 and RHV3 appear to be phylogenetically more distant. This suggests the existence of distinct genogroups within the genus *Batrachovirus* (Figs 15 and 16). The virus has a relatively shorter genome compared to the other members of the genus *Batrachovirus*. Although surprising, the loss of significant portions of the BfHV1 genome secondary to major mistakes in the assembly appears unlikely because 1) the overall arrangement of the genome map, which overlaps with those of the other *Batrachoviruses* (Fig. 14); the multiple confirmatory PCRs carried out across the genome (see above); and above all, the PCR across the proposed genome termini along with the Sanger sequencing of the obtained amplicon, which exclude the loss of long genome portions at either predicted genome ends. Furthermore, all the genes conserved across the *Alloherpesviridae* have been identified among the putatively encoded ORFs in the BfHV1 genome. A number of presumptively immunomodulatory genes have been identified (Table S1) although in smaller numbers compared to those observed in RHV3^17^. However, similar to the common frogs affected by RHV3, common toads infected with BfHV1 also show a relatively rare or inconsistent immune response in the skin lesions. Interestingly, the presence of a detectable infiltrate was rarely observed and only affecting the *stratum basale* of the epidermis, resembling the early lesions of *Chelonid herpesvirus* 5 (ChHV5) associated-papillomatosis^27^. Additionally, in affected toads, moderate to severe hyperkeratosis was observed. This is in contrast to what we observed in RHV3-infected common frogs. The distinct pathology phenotypes in RHV3-infected frogs and BfHV1-infected toads might reflect part of the differences between the RHV3 and BfHV1 genomes together with those concerning the skin anatomy of the affected species, partially justifying the apparent species specificity of both viruses. Interestingly, BfHV1 was not detected in common frogs with proliferative skin disease associated with RHV3 found in a pond known to contain BfHV1-positive toads. Similarly, the toad infected with BfHV1 from the same pond (canton of Neuchatel) were negative for RHV3 despite the presence of RHV3-positive frogs. Furthermore, the presence of both BfHV1 and RHV3 in the same environment might suggest that both viruses require similar conditions to replicate in their respective hosts. Remarkably, and in contrast to RHV3, BfHV1 has been detected in the skin but also in the brain of at least one of the affected toads suggesting a systemic distribution of the virus, which has yet to be proven in RHV3-infected frogs. Furthermore, the presence of BfHV1 was also demonstrated in toads that showed only mild microscopic but no gross skin lesions; suggesting the virus may be more prevalent than what is suggested by the gross findings alone.

Finally, the actual distribution of the virus outside Switzerland is not clear. An anecdotal report describes the occurrence of similar lesions in common toads in Belgium associated with a non-identified herpesvirus^12^. In Germany lesions similar to those that we observed in toads infected with BfHV1 were attributed to a putative fungal infection (Schwärzepilz = Black fungus)^28^. Herpesviruses are commonly considered agents of limited concerns in amphibians^12^. However, the identification of two novel herpesvirus agents in frogs and toads, respectively, in the last two years^17^, is indirect evidence of the relatively poor attention that has been given to these microbes until now. In particular, the apparent “enzootic” nature of BfHV1 infection in the Swiss free ranging toad population along with its wide distribution, likely presence outside Switzerland, and association with mortality calls for a thorough assessment of the role herpesviruses may have for amphibians.

## Electronic supplementary material

Table S1

## Data Availability

All the data concerning this investigation are reported in this article, in the associated supplemental material and in the Genbank database.
