# Supplementary material for: Bufonid herpesvirus 1 (BfHV1) associated dermatitis and mortality in free ranging common toads (Bufo bufo) in Switzerland
Source: Sci Rep. 2018 Oct 3;8:14737. doi: 10.1038/s41598-018-32841-0 (PMC6170376; doi:10.1038/s41598-018-32841-0)
Supplement: Supplementary file 1 — Table S1 [file 41598_2018_32841_MOESM1_ESM.doc]

**Manuscript Title** *Bufonid herpesvirus* 1 (BfHV1) associated dermatitis and mortality in free ranging common toads (*Bufo bufo*) in Switzerland

**Authors: ^1^Francesco C. Origgi*, ^2^Benedikt R. Schmidt, ^3^Petra Lohmann, ^4^Patricia Otten, ^1^Roman K. Meier, ^1^Simone R. R. Pisano, ^1^Gaia Moore-Jones, ^5^Marco Tecilla, ^1^Ursula Sattler, ^1^Thomas Wahli, ^6^Veronique Gaschen, ^6^Michael H. Stoffel**

^1^University of Bern, Centre for Fish and Wildlife Health (FIWI), Department of Infectious Diseases and Pathobiology, Länggassstrasse 122, 3001 Bern, SWITZERLAND; francesco.origgi@vetsuisse.unibe.ch; ^2^Info fauna Karch, UniMail, Batiment G, Bellevaux 51, 2000 Neuchâtel, SWITZERLAND & Department of Evolutionary Biology and Environmental Studies, University of Zurich, Winterthurerstrasse 190, 8057 Zurich, SWITZERLAND; ^3^P. Lohmann, Veterinarian, 8127 Forch, SWITZERLAND; ^4^Fasteris SA, Geneva, SWITZERLAND; ^5^University of Milan, Milan, Italy; ^6^Division of Veterinary Anatomy, University of Bern, POB, Bern, SWITZERLAND

***Corresponding author:** Francesco C. Origgi**,** ^1^University of Bern, Centre for Fish and Wildlife Health (FIWI), Department of Infectious Diseases and Pathobiology**,** Länggassstrasse 122, 3001 Bern, SWITZERLAND; Tel + 41 31 631 2445; Fax + 41 31 631 2635; email: francesco.origgi@vetsuisse.unibe.ch

**Table S1. *Bufonid herpesvirus* 1 (BfHV1) Open reading frames (ORF)** features

| **ORF** | **Herpes or other virus**  **Homologue** | **Protein** | **Predicted function/**  **description** | **Similarity or confidence** | **Position** | **Length (nt)** | **Frame** |
| --- | --- | --- | --- | --- | --- | --- | --- |
| 1 | None | Unknown | Unknown | N/A | 232-777 | 546 | - |
| 2 | None | Unknown | Unknown | N/A | 560-952 | 393 | + |
| 3 | None | Unknown | C-type Mannose receptor-2 | 100%^b^  (40% coverage) | 1,531-2,442 | 912 | - |
| 3a | Muromegalov. WP15B | Unknown | m140 protein | 45%^a^  29/64 | 1,999-2,430 | 432 | + |
| 4 | None | Unknown | Putative C-type lectin protein | 100%^b^  (40% coverage) | 2,550-3,398 | 849 | - |
| 5 | None | Unknown | Unknown | N/A | 3,529-4,434 | 906 | - |
| 5a | None | Unknown | Unknown | N/A | 4,021-4,347 | 327 | + |
| 6 | None | Unknown | Unknown | N/A | 5,157-5,933 | 777 | - |
| 7 | HHV1 | US11 | DNA binding protein | 52%^a^  25/48 | 7,280-10,684 | 3,405 | - |
| 7a | None | Unknown | Transferase | 44.1% ^b^  (59% coverage) | 9,030-9,611 | 582 | + |
| 8 | None | Unknown | Unknown | N/A | 13,193-14,710 | 1,518 | - |
| 9 | CeHV9 | UL20 | Envelope protein | 46%^a^  24/52 | 14,832-16,133 | 1,302 | - |
| 10 | None | Unknown | Allene oxide cyclase-like | 29.2%^b^  (20% coverage) | 17,430-18,482 | 1,053 | - |
| 11 | None | Unknown | DNA/RNA binding protein | 68.9%^b^  (42% coverage) | 18,835-19,212 | 378 | - |
| 12 | Hantavirus | Unknown | Nucleoprotein | 24.4%^b^  (4% coverage) | 19,163-19,837 | 675 | - |
| 13 | EBV | Unknown | gp42 | 99.8%^b^  (56% coverage) | 20,175-20,747 | 573 | - |
| 14 | None | Unknown | Unknown | N/A | 21,714-22,190 | 477 | - |
| 14a | None | Unknown | De novo protein | 52%^b^  (15% coverage) | 21,767-22,282 | 516 | - |
| 14b | None | Unknown | Transport protein | 47.6%^b^  (60% coverage) | 21,835-22,248 | 414 | + |
| 15 | None | Unknown | Hydrolase | 100%^b^  (79% coverage) | 23,993-25,192 | 1,200 | + |
| 15a | None | Unknown | Transcription factor | 31.4%^b^  (33% coverage) | 24,793-25,167 | 375 | - |
| 16 | CyHV-2 | Ribonucleotide reductase-Subunit 2 | De novo DNA synthesis | 217/312^a^  (69%) | 25,176-26,138 | 963 | + |
| 17 | None | Unknown | Isomerase | 100%^b^  (86% coverage) | 26,165-27,211 | 1,047 | + |
| 18 | TeHV3 | TE11 | C-lectin type protein | 70/118^a^  (59%) | 27,370-28,266 | 897 | + |
| 19 | None | Unknown | Lectin-like | 31.9%^b^  (22% coverage) | 28,735-29,043 | 309 | + |
| 20 | CyHV-1 | ORF136a | Signal peptide (immune-regulation)-**CD27-like** | 41/93^a^  (44%) | 28,752-29,753 | 822 | - |
| 21 | None | Unknown | lectin-related NK cell receptor ly49l1 | 100%^b^  (54% coverage) | 29,934-30,593 | 660 | - |
| 22 | None | Unknown | Unknown | N/A | 30,862-32,136 | 1,275 | - |
| 23 | None | Unknown | Unknown | N/A | 32,031-32,510 | 546 | - |
| 24 | None | Unknown | Unknown | N/A | 32,465-34,015 | 1,551 | + |
| 25 | RHV1 | ORF99 | Putative double stranded RNA binding motif | 31/78^a^  (39%) | 34,076-34,564 | 489 | - |
| 26 | RHV1 | ORF95 | Capsid triplex subunit 2 | 169/317^a^  (53%) | 34,599-35,525 | 927 | - |
| 27 | RHV1 | ORF94 | Similar to IcHV-1 ORF26 (RNA bind-prot.) | 68/146^a^  (46%) | 35,506-35,994 | 489 | - |
| 28 | RHV1 | ORF93 | Helicase | 312/531^a^  (58%) | 35,793-37,382 | 1,590 | - |
| 29 | RHV1 | ORF92 | similar to IcHV-1 ORF24 | 117/245^a^  (47%) | 37,382-38,338 | 957 | - |
| 30 | None | Unknown | Unknown | N/A | 38,332-39,150 | 819 | + |
| 31 | None | Unknown | Ribosome-like protein | 61.4%^b^  (26% coverage) | 39,000-39,353 | 354 | + |
| 32 | None | Unknown | Oxidoreductase-like protein | 100%^b^  (coverage 61%) | 39,390-39,662 | 273 | + |
| 33 | None | Unknown | Putative Ras association domain-containing family protein 5 | 29.4%^b^  (9% coverage) | 39,631-40,776 | 1,146 | - |
| 34 | RHV1 | ORF89 | chromosome segregation protein SMC | 202/524^a^  (38%) | 40,877-44,368 | 3,492 | + |
| 35 | CyHV3 | ORF96 | ORF96 (transferase inhibitor) | 24/55^a^  (43%) | 44,292-44,627 | 336 | - |
| 36 | RHV1 | ORF88 | Similar to RaHV-2 ORF122 and IcHV-1 ORF64 | 260/510^a^  (50%) | 44,446-45,963 | 1,518 | - |
| 36a | Unknown | Unknown | Lyase | 28^b^  (8% coverage) | 45,089-45,433 | 345 | + |
| 37 | RHV1 | ORF87 | Helicase-primase subunit | 309/683^a^  (45%) | 45,927-48,074 | 2,184 | - |
| 38 | RHV1 | ORF86 | DNA (cytosine-5-)-methyltransferase | 393/740^a^  (53%) | 48,129-50,426 | 2,298 | + |
| 39 | RHV1 | ORF42 | putative ATPase subunit of terminase | 191/264 ^a^ (72%) | 50,273-51,247 | 951 | - |
| 40 | RHV1 | ORF85 | Similar to IcHV-1 ORF61 | 126/284^a^  (44%) | 51,246-52,196 | 951 | + |
| 41 | RHV1 | ORF84 | similar to RaHV-2 ORF118 and IcHV-1 ORF60 | 222/428^a^  (51%) | 52,254-53,483 | 1,230 | - |
| 42 | RHV1 | ORF83 | multiple membrane-spanning protein | 104/173^a^  (60%) | 53,485-54,207 | 723 | + |
| 43 | RHV1 | ORF82 | multiple membrane-spanning protein | 72/165^a^  (43%) | 54,324-54,932 | 609 | - |
| 44 | RHV2 | ORF115 | multiple membrane-spanning protein | 38/87^a^  (43%) | 54,942-55,322 | 381 | - |
| 45 | Unknown | Unknown | Unknown | N/A | 55,630-56,160 | 531 | + |
| 46 | RHV1 | ORF76 | capsid triplex protein 1 | 116/273^a^  (42%) | 56,157-57,806 | 1,650 | - |
| 46a | Unknown | Unknown | Unknown | N/A | 56,726-57,034 | 309 | - |
| 46b | Unknown | Unknown | Unknown | N/A | 57,382-57,657 | 276 | + |
| 47 | RHV1 | ORF75 | similar to RaHV-2 ORF113 and IcHV-1 ORF54 | 292/570^a^  (51%) | 57,817-59,454 | 1,638 | + |
| 48 | RHV1 | ORF74 | probable similar to RaHV-2 ORF112 and IcHV-1 ORF55 | 176/383^a^  (45%) | 59,369-60,601 | 1,233 | + |
| 49 | RHV1 | ORF73 | similar to RaHV-2 ORF111 and IcHV-1 ORF56 | 675/1234^a^  (54%) | 60,655-64,401 | 3,747 | - |
| 49a | None | Unknown | Unknown | N/A | 61,932-62,210 | 279 | + |
| 49b | None | Unknown | Unknown | N/A | 62,738-63,112 | 375 | + |
| 50 | RHV1 | ORF72 | DNA polymerase | 843/1480^a^  (56%) | 64,356-68,672 | 4,317 | + |
| 50a | None | Unknown | Transcription motif | 24%^b^  (28% coverage) | 64,425-64,769 | 345 | - |
| 50b | None | Unknown | Ferrodoxin-like | 39%^b^  (37% coverage) | 65,280-65,576 | 297 | - |
| 50c | None | Unknown | Unknown | NA | 65,501-65,806 | 306 | - |
| 50d | None | Unknown | DNA repair motif | 58%^b^  (34% coverage) | 67,562-67,843 | 282 | - |
| 51 | None | Unknown | Immunoglobulin-like beta-sandwich motif | 15.1%^b^  (40% coverage) | 68,673-68,954 | 282 | - |
| 52 | None | Unknown | Putative transcription factor | 49.6%^b^  (29% coverage) | 68,953-69,414 | 462 | + |
| 53 | RHV1 | ORF69 | Possible member of PK gene family | 48/115^a^  (41%) | 69,415-72,456 | 3,042 | - |
| 53a | Unknown | Unknown | Unknown | NA | 71,626-71,895 | 270 | + |
| 54 | RHV1 | ORF68 | Protein Kinases, catalytic domain | 229/528^a^  (43%) | 72,456-74,651 | 2,196 | - |
| 55 | RHV1 | ORF67 | Predicted signal peptide | 239/482^a^  (49%) | 74,641-76,752 | 2,112 | + |
| 55a | None | Unknown | Unknown | NA | 76,368-76,691 | 324 | + |
| 56 | RHV1 | ORF66 | member of ORF24 gene family-predicted signal peptide | 188/327^a^  (57%) | 76,771-77,880 | 1,110 | + |
| 57 | RHV1 | ORF65 | Zinc-RING-finger protein | 137/354^a^  (38%) | 77,916-79,727 | 1,812 | - |
| 58 | RHV1 | ORF65 | Zinc-RING-finger protein | 182/438^a^  41.5% | 79,858-81,909 | 2,052 | - |
| 59 | RHV1 | ORF63 | putative capsid maturational protease | 282/511^a^  (55%) | 82,207-84,054 | 1,848 | - |
| 60 | None | Unknown | Unknown | NA | 84,008-84,595 | 588 | - |
| 61 | HHV1 | UL31 | Putative virion egress molecule | 50.6%^b^  (22% coverage) | 84,351-85,193 | 843 | + |
| 62 | RHV1 | ORF59 | Hypothetical Protein | 215/363^a^  (59%) | 85,182-86,285 | 1,104 | - |
| 63 | RHV1 | ORF58 | similar to IcHV-1 ORF43 (putative isomerase) | 404/828^a^  (48%) | 86,284-88,884 | 2,601 | + |
| 63a | None | Unknown | Unknown | N/A | 86,709-87,386 | 678 | - |
| 63b | None | Unknown | Unknown | N/A | 88,391-88,663 | 273 | - |
| 64 | RHV1 | ORF57 | Hypothetical protein | 110/277^a^  (39%) | 88,881-89,738 | 858 | - |
| 65 | Unknown | None | Unknown | 43%^a^  280/641 | 89,802-96,137 | 6,336 | + |
| 65a | None | Unknown | Unknown | N/A | 90,650-91,045 | 396 | - |
| 65b | None | Unknown | Unknown | NA | 92,186-92,707 | 522 | - |
| 66 | RHV1 | ORF55 | similar to RaHV-2 ORF81 | 131/226^a^  (57%) | 96,201-97,007 | 807 | - |
| 67 | None | Unknown | Hydrolase | 33.3%^b^  (22% coverage) | 96,804-97,121 | 318 | + |
| 68 | RHV1 | ORF54 | Major capsid protein | 738/1330^a^  (55%) | 97,013-100,930 | 3,918 | - |
| 69 | None | Unknown | Apopotosis-associated molecule (putative) | 28.1%^b^  (21% coverage) | 100,896-101,564 | 669 | - |
| 70 | RHV1 | ORF52 | Similar to RaHV-2 ORF78 and IcHV-1 ORF37 | 324/622^a^  (52%) | 101,479-103,404 | 1,926 | + |
| 70a | None | Unknown | Transcription/transcription activator | 16%^b^  (31% coverage) | 103,095-103,397 | 303 | - |
| 71 | RHV1 | ORF50 | similar to RaHV-2 ORF76; similar to IcHV-1 ORF35 | 75/175^a^  (42%) | 103,401-103,838 | 438 | - |
| 72 | None | Unknown | Unknown | N/A | 103,835-104,413 | 579 | - |
| 72a | None | Unknown | Oxydoreductase-like | 24.8%^b^  (29% coverage) | 104,023-104,466 | 444 | + |
| 73 | RHV1 | ORF49 | similar to RaHV-2 ORF75 and IcHV-1 ORF34 | 274/450^a^  (60%) | 104,262-105,617 | 1,356 | - |
| 74 | None | Unknown | Delta-endotoxin-like | 52.2%^b^  (23% coverage) | 105,461-106,126 | 666 | - |
| 75 | None | Unknown | Unknown | N/A | 105,975-106,514 | 540 | + |
| 76 | RHV1 | ORF47 | membrane glycoprotein | 103/203^a^  (50%) | 106,514-107,506 | 993 | + |
| 77 | RHV1 | ORF46 | Membrane glycoprotein | 667/1161^a^  (57%) | 107,352-110,897 | 3,546 | - |
| 77a | None | Unknown | Putative glycoprotein | 9.5%^b^  (44% coverage) | 107,584-107,877 | 294 | + |
| 77b | None | Unknown | Unknown | N/A | 109,136-109,564 | 429 | + |
| 78 | RHV1 | ORF45 | membrane glycoprotein | 216/394^a^  (54%) | 110,953-112,164 | 1,212 | - |
| 79 | RHV1 | ORF44 | similar to RaHV-2 ORF70; similar to IcHV-1 ORF68 | 155/340^a^  (45%) | 112,052-113,128 | 1,077 | + |
| 80 | RHV1 | ORF42 | Terminase | 123/174^a^  (70%) | 113,375-113,902 | 528 | + |
| 81 | RHV1 | ORF43 | similar to RaHV-2 ORF69 and IcHV-1 ORF70 | 101/198^a^  (51%) | 113,808-114,665 | 858 | - |
| 82 | RHV1 | ORF42 | Terminase | 156/269^a^  (57%) | 114,569-115,366 | 798 | + |
| 82a | None | Unknown | Hydrolase-like motif | 60.9%^b^  (31% coverage) | 114,778-115,101 | 324 | - |
| 83 | None | Unknown | GIY-YIG endonuclease | 97.6%^b^  (18% coverage) | 115,333-116,721 | 1,389 | + |
| 84 | RHV1 | ORF40 | Thymidine Kinase-like | 106/221^a^  (47%) | 116,822-117,472 | 651 | + |
| 84a | None | Unknown | Actin-binding protein | 29.9%^b^  (51% coverage) | 117,052-117,333 | 282 | - |
| 85 | None | Unknown | Unknown | N/A | 117,884-118,525 | 642 | - |
| 86 | Rhesus rotavirus | VP4 | Sialic acid binding domain | 33.1%^b^  (17% coverage) | 118,600-118,977 | 378 | - |
| 87 | RHV1 | ORF35 | member of ORF35 gene family | 150/393^a^  (38%) | 118,995-120,158 | 1,164 | - |
| 88 | None | Unknown | Unknown | N/A | 120,159-122,309 | 2,151 | - |
| 89 | RHV1 | ORF34 | Major outer envelope glycoprotein | 135/362^a^  (37%) | 122,477-124,132 | 1,656 | - |
| 90 | RHV1 | ORF67 | Predicted signal peptide | 187/467^a^  (40%) | 124,140-126,656 | 2,517 | - |
| 90a | None | Unknown | Unknown | NA | 124,159-124,443 | 285 | + |
| 90b | None | Unknown | Unknown | N/A | 125,746-126,030 | 285 | + |
| 91 | RHV1 | ORF33 | Predicted protein (putative transcription regulator) | 58/145^a^  (40%) | 126,681-128,069 | 1,389 | - |
| 92 | None | Unknown | Unknown | N/A | 128,087-129,313 | 1,227 | - |
| 93 | None | Unknown | Unknown | NA | 129,312-130,496 | 1,185 | + |
| 94 | RHV1 | ORF30 | Member of ORF30 gene family | 159/385^a^  (41%) | 130,507-131,646 | 1,185 | + |
| 94a | None | Unknown | Unknown | N/A | 130,866-131,135 | 270 | - |
| 95 | None | Unknown | Putative short chain cytokine motif | 30.7%^b^  (16% coverage) | 131,847-132,170 | 324 | + |
| 96 | None | Unknown | Putative calcium-binding protein | 21.3%^b^  (16% coverage) | 132,167-132,445 | 279 | + |
| 97 | None | Unknown | tumor necrosis factor receptor superfamily member 10a | 99.6%^b^  (77% coverage) | 132,174-132,479 | 306 | - |
| 98 | RHV1 | ORF26 | similar to RaHV-2 ORF95; similar to IcHV-1 ORF75 | 159/317^a^  (50%) | 132,739-133,665 | 927 | - |
| 98a | None | Unknown | Isomerase | 31.4%^b^  (34% coverage) | 133,133-133,576 | 444 | + |
| 99 | RHV1 | ORF25 | Predicted Zn-binding protein | 167/337^a^  (49%) | 133,742-134,734 | 993 | + |
| 100 | None | Unknown | Chelatase-like | 38.6%^b^  (29% coverage) | 134,805-135,317 | 513 | + |
| 101 | None | Unknown | Unknown | N/A | 135,115-137,901 | 2,787 | + |
| 102 | RHV1 | ORF24 | Predicted signal peptide | 156/358^a^  (43%) | 137,888-139,120 | 1,233 | + |
| 103 | None | Unknown | Unknown | N/A | 139,184-139,807 | 624 | - |
| 104 | TeHV3 | UL17 | Tegument protein | 35/75^a^  (46%) | 139,862-140,737 | 876 | - |
| 105 | None | Unknown | Unknown | N/A | 141,709-142,488 | 780 | - |
| 106 | None | Unknown | Unknown | N/A | 142,593-143,438 | 846 | - |
| 107 | None | Unknown | Unknown | N/A | 143,489-143,776 | 288 | - |
| 108 | None | Unknown | Unknown | N/A | 143,556-144,599 | 1,044 | - |
| 109 | None | Unknown | Unknown | N/A | 144,766-145,857 | 1,092 | + |
| 110 | None | Unknown | Hydrolase | 95.2%^b^  (14% coverage) | 146,154-148,283 | 2,130 | + |
| 110a | AngHV1 | ORF34 | FAM75 family | 54/97^a^  (55%) | 147,211-147,657 | 447 | + |
| 111 | Murine RV | ORF67 | Capsid maturation protease (complement c3-like) | 39/71^a^  (54%) | 148,521-149,216 | 696 | + |
| 112 | None | Unknown | Transferase-like | 18.8%^b^  (52% coverage) | 149,580-149,897 | 318 | - |
| 113 | PaHV-2 | Unknown | Putative transcriptional regulator motif (ICP4) | 19/33^a^  (57%) | 149,984-152,317 | 2,334 | - |
| 113a | None | Unknown | Unknown | N/A | 150,792-151,088 | 297 | + |
| 113b | None | Unknown | Unknown | N/A | 151,543-152,139 | 597 | - |
| 114 | None | Unknown | Unknown | N/A | 152,744-153,028 | 285 | - |
| 115 | LCV Macaca | BPLF1 | Predicted tegument protein (Ph2 transc. Regulator c.97.1%-Cov.13%) | 87/195^a^  (44%) | 153,125-155,725 | 2,601 | + |
| 115a | None | Unknown | Unknown | N/A | 153,737-154,063 | 327 | - |
| 115b | None | Unknown | Unknown | N/A | 155,111-155,620 | 510 | - |
| 116 | None | Unknown | SipA N-terminal domain-like | 71.2%^b^  (21% coverage) | 156,778-157,230 | 453 | - |

^a^The number at the denominator indicates the total length of the portion of the herpesviral (or other virus) homologous protein being compared by BLAST (<http://blast.ncbi.nlm.nih.gov/Blast.cgi>) with BfHV1. The number at the numerator indicates the actual number of the amino acid residues of BfHV1 showing similarities with the compared portion of the homologous protein. The percentage (in parenthesis) summarizes the overall similarity between BfHV1 and the specific herpesvirus homologous protein for the specific motif considered.

^b^The value outside parenthesis refers to the confidence (probability) of an actual structural homology existing between either the entire or part of the predicted encoded protein by BfHV1 and that detected by Phyre2 software (http://www.sbg.bio.ic.ac.uk/~phyre2/html/page.cgi?id=index), whereas the value indicated in parenthesis refers to the coverage of the predicted protein that the confidence value is referred to.
